# Testing the Sandblasting Process in the Manufacturing of Reference Spheres for Non-Contact Metrology Applications

**DOI:** 10.3390/ma14185187

**Published:** 2021-09-09

**Authors:** Víctor Meana, Eduardo Cuesta, Braulio J. Álvarez

**Affiliations:** Departamento de Construcción e Ingeniería de Fabricación, Universidad de Oviedo, Calle Pedro Puig Adam, E.D.O.5, 33203 Gijon, Asturias, Spain; meanavictor@uniovi.es (V.M.); braulio@uniovi.es (B.J.Á.)

**Keywords:** sandblasting, precision spheres, non-contact metrology, laser scanning, laser sensors

## Abstract

To ensure that measurements can be made with non-contact metrology technologies, it is necessary to use verification and calibration procedures using precision artefacts as reference elements. In this environment, the need for increasingly accurate but also more cost-effective calibration artefacts is a clear demand in industry. The aim of this work is to demonstrate the feasibility of using low-cost precision spheres as reference artefacts in calibration and verification procedures of non-contact metrological equipment. Specifically, low-cost precision stainless steel spheres are used as reference artefacts. Obviously, for such spheres to be used as standard artefacts, it is necessary to change their optical behavior by removing their high brightness. For this purpose, the spheres are subjected to a manual sandblasting process, which is also a very low-cost process. The equipment used to validate the experiment is a laser triangulation sensor mounted on a Coordinate Measuring Machine (CMM). The CMM touch probe, which is much more accurate, will be used as a device for measuring the influence of sandblasting on the spheres. Subsequently, the influence of this post-processing is also checked with the laser triangulation sensor. Ultimately, the improvement in the quality of the point clouds captured by the laser sensor will be tested after removing the brightness, which distorts and reduces the quantity of points as well as the quality of the point clouds. In addition to the number of points obtained, the parameters used to study the effect of sandblasting on each sphere, both in contact probing and laser scanning, are the measured diameter, the form error, as well as the standard deviation of the point cloud regarding the best-fit sphere.

## 1. Introduction

**Figure 1:**
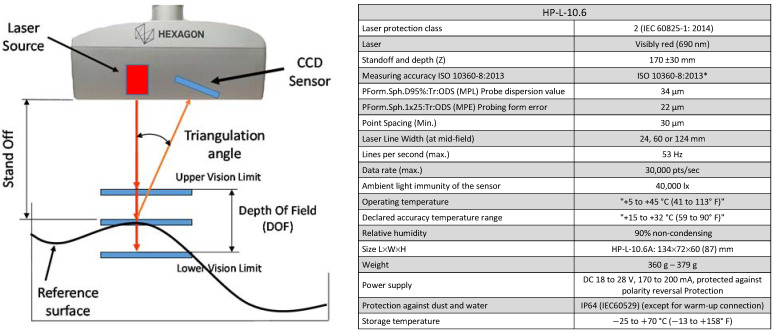
Principle of laser triangulation scanning.

Metrological verification using optical equipment is of increasing interest in industry. In this sense, one of the most widespread technologies is laser triangulation sensors, either mounted on coordinate measuring arms or on tridimensional coordinate measuring machines, or even as independent sensors that can be integrated in multiple industrial applications. When a laser triangulation sensor is used for scanning (Figure 1), a laser beam is first projected onto the surface to scan, then the projected intersection line is captured by a digital camera, and finally the coordinates of the points are determined by trigonometric calculations (hence the term “triangulation”), taking into account the angle between the laser beam and the camera orientation, and the light intensity captured by the digital camera, among other parameters [1]. The highly extended deployment of these sensors has also been possible due to improvements in the accuracy and capabilities of these devices. Manufacturers have enhanced these instruments through adjustment and calibration processes, apart from increasing the quality of designs and materials for the internal components. Currently, many users and researchers employ these sensors for measurement (Geometrical Dimensional and Tolerancing - GD&T- verification) as well as for reverse engineering typical tasks.

The improvement in accuracy, including traceability assessment [2,3,4], is a crucial factor to consider. It is precisely in this line of research where several factors that have an influence on laser triangulation sensors have been analyzed. Apart from the equipment’s own parameters and specific tests [1,5,6], other parameters have also been taken into account, such as scanning speed [7] and scanning strategy [8,9]. Moreover, external parameters, like those derived from the part or object acting as the measurand, such as the material [10,11], color, surface roughness [12,13], ambient light [14], or even the type of the geometry to scan [3,15,16]. The idea is to assess the measurements that can be made with these non-contact technologies, thus extending their application beyond typical reverse engineering applications to metrological (GD & T inspection) ones.

Two of the fundamental parameters in the scanning quality of this type of sensors are the orientation of the sensor with regard to the target surface [8,17] and the surface finish of the part [12,18,19]. Shiny surfaces cause defects in the captured pointclouds, whereas, on the contrary, matte surfaces enhance the coverage of the capture. To achieve this type of surface finish over metallic surfaces, sandblasting is one of the most promising processes. In fact, for certain optical instruments, sandblasted surfaces are among the few surfaces that allow a measurement accuracy comparable to that achieved by contact measurement [18].

The aim of this work is to validate the sandblasting process as a process for modifying the surface condition of precision spheres in order to use these spheres as reference artefacts for adjustment, verification and/or calibration of optical sensors and non-contact reverse engineering equipment. Only if this process is sufficiently conservative with respect to the geometry of the precision spheres can it be valid for creating reference objects. On the contrary, if the damage or modification (dimensional or geometrical) is excessive, the process will not be suitable for this purpose.

In particular, a laser triangulation sensor (3D scanning) mounted on a Coordinate Measuring Machine (CMM) was used in this study. The CMM permits automation of the scanning process, avoiding errors derived from manual operation (as occurs with laser triangulation sensors mounted on coordinate measuring arms). Thus, factors like focal distance, scanning orientation, or scanning density were not considered as variables that can influence the experiment.

Although the finishing process is identical, the essential difference with respect to the work presented in [19] is that in that work the geometrical quality of the workpieces was qualified as very low, with a high surface roughness. The spheres were manufactured by metal laser sintering (SLM), and the post sandblasting was aimed at improving the geometrical quality, which was proved by both contact and laser measurements. In fact, the improvement ascertained by the contact measurement was very high.

However, the situation is the opposite in the case presented here. The original workpieces possess a high dimensional and geometrical accuracy, with very low deviations for the diameter and sphericity, due to the application of superfinishing processes and a final polishing. Conversely, this excellent surface finish makes it very difficult (and can even impede) the measurement of these spheres with laser triangulation sensors. By sandblasting these spheres, the purpose is to enable their use in laser measurement, but assuming a probable and unavoidable loss of accuracy caused by sandblasting.

Specifically, the objective of this article is, on one hand, to quantify the loss of dimensional and/or geometrical accuracy, and, on the other hand, to quantify the improvement in the laser capturing. The sandblasted spheres are suitable as reference elements only when the loss of accuracy is not too high and, on the contrary, when the improvement in laser capturing is substantial.

The final target of this research is to find out whether a low-cost process (manual sandblasting) can be applied to stainless steel precision spheres, of very low cost as well, to materialize calibration spheres for non-contact metrology. It is expected that the loss of precision in both diameter and form error of the post-blasting spheres will be low enough for this purpose. Ideally, the form errors of the sandblasted spheres should be at least one order of magnitude lower than the measurement uncertainty of the optical equipment. In any case, this experimentation will reveal which equipment is suitable for being calibrated with this type of sandblasted spheres.

Nowadays, the manufacturing of precision ceramic spheres (grades G3, G5 or G10, with sphericity < 0.25 µm, *Ra* < 0.020 µm, according to ISO 3290/DIN 5401 [20]) is very costly. This is mainly due to the fact that they are built specifically for this purpose, starting from ceramic powder, which is sintered and subsequently polished. They also require high hardness and wear resistance, using materials such as ruby, alumina, sapphire, or zirconia, among others. However, when they are intended to be used as reference elements for non-contact measurements, neither high hardness nor high wear resistance is required. In the context of the aims presented in this work, it is in fact sufficient that they are made of stainless materials, for example aluminum alloys or steels of qualities such as AISI 304, AISI 306L, or similar.

The spheres used in this research are stainless steel precision balls commonly used in the bearing industry, the cost of which is lower, but which also feature worse manufacturing qualities (G50 or G100 [20], with sphericity < 2.5 µm, *Ra* < 0.1 µm). 

For optical applications, G100 accuracy or lower (according to ISO 3290 [20]) is enough unless the spheres show excessive brightness. Specifically, the idea of the experiment is to eliminate the very shiny finish (mirror-like) of the sphere surface, checking the variations in both diameter and form error caused by the sandblasting process. Another very important objective of the experiment is to quantify (if it exists) the improvement in the quality of the point clouds achieved by a laser triangulation equipment with respect to the cloud obtained on the polished, pre-sanded sphere.

The shot peening process, in this case sandblasting, will be carried out in a manual sandblasting machine using sand with fine grain size. Obviously, a certain variability is introduced even though the process variables are controlled (grain size, exposure time, distance, and the incident direction of the sandblasting stream onto the spheres). This variability must be studied so that the detected wear (or surface attack) will be assessed as rigorously and objectively as possible.

Therefore, the work includes sandblasting tests on stainless steel spheres of different diameters, evaluating (by CMM measurement) the variation of the mean diameter value and especially the loss of form error. In addition, it will also be interesting to analyze the variation of the standard deviation of the point cloud, as this is a crucial parameter in the measurement of the quality of the laser point cloud [19].

## 2. Materials and Methods

Since the process chosen to perform the modification of the surface condition is manual sandblasting, it requires statistical validation to be considered valid, minimizing the operator’s influence on a given sphere. Therefore, the experimentation includes a range of different sets of spheres, each set corresponding to a different nominal diameter.

**Figure 2:**
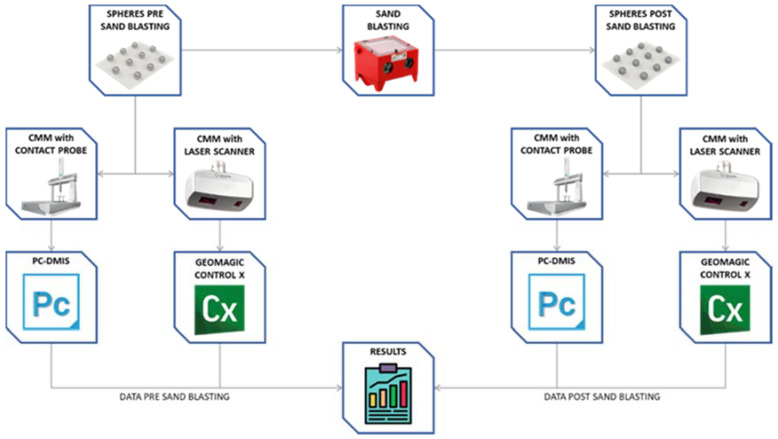
Research methodology.

Specifically, 3 sets of spheres of different size were analyzed (3 plates including 10 spheres each, of Ø 10, Ø 18 and Ø 25 mm, respectively, as shown in Figure 2). From these analyses, the average values and standard deviations are determined for each one of the sets of 10 spheres, which are distributed over the corresponding plate with a similar layout. The diameter and form error values of the spheres of each set have been measured by contact (CMM with SP25M scanning probe) and by laser triangulation (Hexagon HP-L-10.6 sensor mounted on the CMM), first at their original polished state, before the surface attack, and secondly after being surface treated by sandblasting.

Contact measurements of the spheres were carried out with a coordinate measuring machine (CMM), the DEA Global Image 091508 model, equipped with a PH10MQ indexing head. A Renishaw SP25 contact scanning probe can be mounted on this head. PC-DMIS 2018 R2 software was used to configure the CMM, setting parameters, paths, and number and distribution of points. The tip used was a Ø 1.5 mm ruby sphere for both polished and post-sanded spheres. The accuracy of this CMM is given by the manufacturer (Hexagon Metrology) according to ISO 10360-2 [21] and according to the latest calibration: $E_{0.\mathrm{MPE}}=2.2+0.003\times L\;\left(µm\right)$, $R_{0.\mathrm{MPL}}=2.2\;µm$.

Although this MPE parameter is not a substitute for uncertainty of dimensional or form measurement, the use of a sufficiently representative number of spheres, as well as several repetitions (at least 3 for each sphere), provides enough traceability of the measurements. This is especially true when working with calibrated equipment and obtaining average values.

For the non-contact measurement, a laser triangulation sensor from Hexagon Metrology, HP-L-10.6, was used, also attached to the CMM. This sensor comes with a calibration certificate (ISO 10360-8) [2] with a maximum error specification of 0.020 mm. On the other hand, the surface treatment of the spheres was carried out with the Sablex S-2 machine using WFA F100 alumina oxide (average grain size 106~150 µm, and true density 3.9 g/cm^3^) as an abrasive element, projected onto the sphere surface at a pressure of 4 bar. Despite being a manual process, the distance of the nozzle was kept in the range of 200–300 mm, with 5 orientations per plate (one orientation normal to the plate, and the other 4 at 45° with respect to the normal vector to the plate, distributed in 4 quadrants). The sandblasting operation time, for all orientations, was inferior to 1 min per plate.

Figure 2 shows a diagram illustrating the methodology followed and the equipment used for the development of the research.

The steps followed in the procedure were:

1. Manufacture of sphere plates. Three sets of rectangular plates were manufactured so that 10 spheres of the same diameter are mounted on each plate. The layout of the spheres of each plate makes handling easy and allows the univocal identification of each sphere inside. The base plates were also made of stainless steel.
2. Contact measurement. The 30 spheres were measured with the CMM obtaining reference values, both dimensional and geometrical, with high accuracy.
3. Non-contact measurement. All 30 spheres were digitalized using non-contact measurement techniques by means of a laser triangulation sensor controlling the power in real time and automatically.
4. Surface treatment of the samples. The surface condition of the spheres was modified by means of a sandblasting process with alumina oxide projection, obtaining sets with less brightness and a different texture.
5. Contact and non-contact measurement of the treated sets. CMM measurements were repeated for the post-sandblasted spheres, both by contact (post-sandblasting reference measurements) and non-contact with the laser triangulation sensor.
6. Analysis of results. The values of the measurements obtained from the contact measurements of the spheres before and after the sandblasting process were compared, as well as the point clouds obtained in both cases from the non-contact measurements.

### Test Specimens Manufacturing and Justification of Material Selection

The material selected for the test plates is AISI 316L stainless steel. AISI 316L was an austenitic stainless steel with <0.03%C, 2%Mn, 17%Cr, 13%Ni, 3%Mo (EN symbol: X2CrNiMoN-17-14-3), with hardness HB < 215 and CTE = 14 × 10^−6^ K^−1^. The plates were also sandblasted prior to any measurement to avoid reflections on the spheres. The precision spheres were drilled using two hemispherical jaws, so that the drill bit did not produce any permanent marks or deformations. Each hole in the sphere was then threaded in order to screw the sphere onto the base plate. All spheres are made of AISI 316L of commercial grade G100 quality, with a sphericity lower than 2.5 µm and an arithmetic mean roughness *Ra* < 0.1 µm.

**Figure 3:**
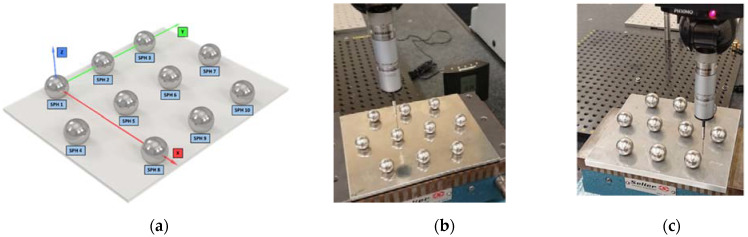
Plate with 10 spheres of the same diameter: (**a**) designation of the spheres and reference system; (**b**) CMM contact measurement of a set of ten spheres (25 mm diameter) on the original base plate; (**c**) CMM contact measurement of a sandblasted base plate.

Figure 3a shows the 3-4-3 matrix design of each set of 10 spheres. This arrangement allows easy sandblasting of each sphere without influence from the adjacent sphere. In addition, it also facilitates the access of the laser triangulation sensor beam (especially above the equator) as well as the contact probe. The designations of the 10 spheres of each set and the coordinate axes of the reference system used for the measurement procedure are also presented in Figure 3a.

The coordinate system of each plate is defined from 3 spheres in such a way that it is independent of the supporting plate. According to its nomenclature (Figure 3a), the coordinate system is formed by spheres 1, 3 and 8 (*XY* plane), with sphere 1 being the origin and sphere 3 defining the *Y* axis. The same alignment definition has been applied for each of the 3 plates with spheres of Ø 10, Ø 18 and Ø 25 mm. Figure 3b shows a detail of the CMM contact measurement (pre-sandblasting) on Ø 25 mm spheres set.

## 3. Results

### 3.1. Pre-Sandblasting Measurements

**Figure 4:**
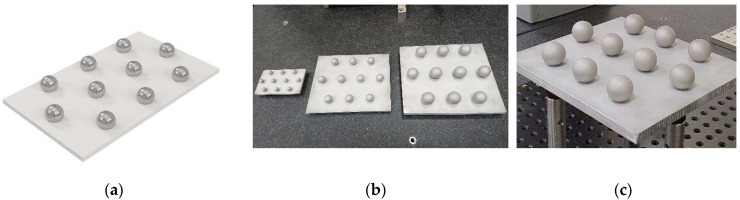
Sphere sets: (**a**) plate of 10 pre-sandblasted spheres (only the support plate has been sandblasted to avoid reflections); (**b**) general view of the 3 plates of 10, 18 and 25 mm diameter spheres; (**c**) detail of the plate with 25 mm post-sandblasting spheres.

During the non-contact measurement of the spheres in their original state (pre-sandblasting) reflections caused by the brightness of the base plate were observed (Figure 3b). These reflections added glare and reduced the quality of the point cloud captured by the laser sensor. This made it necessary to sandblast the base plate (Figure 3c and Figure 4a), prior to the insertion of the pre-blasted spheres. Once the pre-blasted spheres had been measured by contact (CMM) and non-contact (laser), the spheres were sandblasted. A uniform and completely matt finish was obtained in the three sets (Figure 4b,c).

Contact measurements were carried out with the SP25 head with a Ø 1.5 mm diameter ruby tip. The laboratory has an air conditioning system that maintains the temperature within 20 ± 1 °C. Measurements were performed on the three sets of spheres with a minimum scanning density of 1 point/mm^2^ for each of the hemispheres (only the upper half of each sphere is measured) of diameters of 10, 18 and 25 mm, respectively. 

Table 1 shows the results of the contact measurements of the spheres in their original surface state before the sandblasting process. These data constitute the reference values for the subsequent comparative analysis that will be carried out with the measurements after sandblasting. Regarding contact measurements, the dimensions evaluated are the diameters of the spheres, the form error, and the standard deviation of the point cloud regarding the best-fit sphere. The average diameter values were 10.0026 mm for the Ø 10 mm spheres, 17.9977 mm for the Ø 18 mm spheres, and 25.0049 mm for the Ø 25 mm spheres, while the average form deviation was 0.0035 mm, 0.0023 mm, and 0.0028 mm, respectively. In contact measurement, the standard deviation parameter (Std. Dev.) tends to be close to 0 (in the higher case, it is lower than the CMM probing error: 0.002 mm). In general terms, these results correspond correctly to the estimated accuracy G100 for stainless steel spheres.

**Figure 5:**
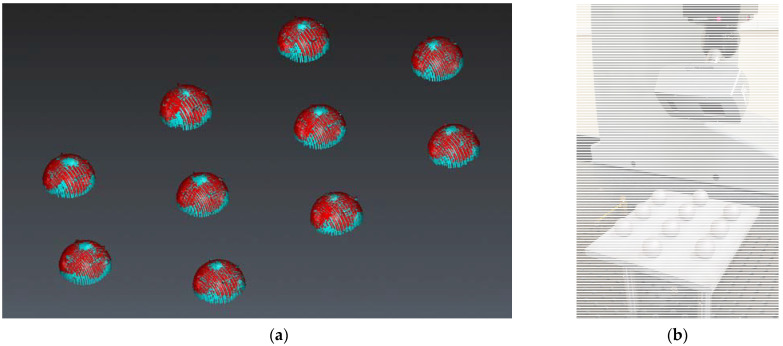
Laser scanning of the spheres: (**a**) point clouds captured at normal gain (red) and high gain (blue), both in pre-sanded state; (**b**) general view of the HP-L-10.6 laser sensor scanning at 45°.

The spheres were then measured using a laser triangulation sensor (HP-L-10.6 from Hexagon Metrology) assembled in the CMM (Figure 5b). To obtain a real and accurate comparison with the contact measurements, all measurements have been carried out under the same environmental conditions (light and temperature) and with the same procedure of alignment and sequence of the spheres. For the sphere captures, five orientations were used (four at 45° from the cardinal points and one from the vertical position, at 0°). These orientations were sufficient to capture at least the upper hemisphere of each sphere. The software used to capture the point clouds is the same PC-DMIS that controls the CMM, although Geomagic Control X software was preferred for point cloud processing, which involved the removal of points belonging to the base plate and those located below the equator of the spheres. Finally, a standard “2·Sigma” filter was applied to this trimmed cloud (hemisphere) to remove spurious points, clearly far from the spheres, which would distort all measurements.

The results obtained are also shown in the central area of Table 1. In addition to the diameter and the form deviation, the value of the standard deviation has been obtained when the cloud is fitted to a best-fit sphere (Least-Squares fitting). The average standard deviations are significantly larger than in the case of contact measurement, which corroborates the idea that a bright surface finish (these are “mirror polished” spheres) is not suitable for being captured with optical equipment. Note (Table 1) that both diameter and form deviation values are far from the reference values (even up to −0.144 mm for Ø 25 mm spheres or 0.187 mm for Ø 10 mm spheres).

On the other hand, the standard deviation of the values obtained by non-contact measurement on polished spheres (original state) reaches high values, on the order of 0.031 mm, regardless of the diameter value. These data show the problems that arise when non-contact measurement systems are used on polished parts because the surface brightness causes the generation of point clouds with poor metrological quality (Figure 5a).

Furthermore, laser measurements on the original spheres had to be carried out in high-gain mode (less sensitivity), because in normal-gain mode (high sensitivity), the sensor was not able to capture enough points, generating very poor clouds. As shown in Figure 5, due to the high brightness of the pre-sanded spheres, point clouds captured with normal gain (red cloud) cover the spheres very poorly, while the coverage is much higher with high gain (blue cloud). This effect is accentuated for the smaller spheres. In fact, in the case of 10 mm spheres, it was not possible to obtain accurate diameter values due to insufficient data. Thus, this high-gain mode allows the comparison between the pre- and post-sandblasting states.

### 3.2. Sandblasting and Post-Sandblasting Measurement

In accordance with the main objective of this work, the surface sandblasting treatment of the test samples was carried out using WFA F100 alumina oxide as the abrasive. The Sablex S-2 blasting machine was programmed to work at a constant pressure of 4 bar. 

**Figure 6:**
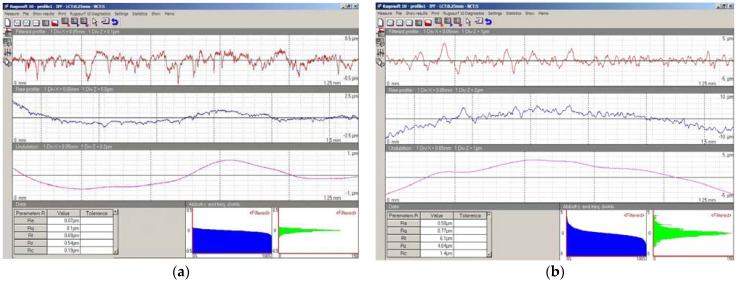
An example of the effect of sandblasting process in the roughness profile and parameters: (**a**) pre-sandblasted Ø 25 mm sphere, original G100; (**b**) post-sandblasted Ø 25 mm sphere.

The roughness of the post-sandblasting spheres was measured with a contact roughness tester (TESA Rugosurf10^®^), and average values of *Ra* = 0.5~0.6 µm were obtained (with the original roughness values being *Ra* < 0.1 µm). As an example, Figure 6 shows a roughness profile and its main parameters measured by the profilometer. Several roughness measurements were performed showing that the variability of *Ra* between each of the ten spheres was low, and independent of the position of the spheres on the plate. This confirms that the finishing achieved with the sandblasting process was adequate and repeatable.

From the geometrical and dimensional point of view analysis, and in the case of post-sandblasting, the measurements made by contact with the CMM machine show higher values for both the diameter of the spheres and the form deviation (Table 2). Please note that the sandblasting process generates a dimensional deviation with an average value of only 2.7 µm and an increase in the form deviation of about 1.7 µm. At this point, it should be taken into account that this value is even lower than the maximum permissible error of the CMM, which is around 2.2 µm. It can therefore be concluded that, apart from a minimal variation in diameter, sandblasting also left the form deviation of the spheres practically unchanged.

The values obtained in the non-contact laser triangulation measurement were generated, as in the pre-sanding stage, using the capture mode with low sensitivity (high gain). The same type of spurious point filter was then applied, although now these defects appeared to a much lesser extent. In this case, the number of points captured with the laser sensor increased for all of the three ranges of spheres, being 13,975 points for each of the Ø 10 mm, 43,052 points for each of the Ø 18 mm, and 80,126 points for each of the Ø 25 mm spheres (Table 3). The increase with respect to the pre-sanded spheres reached 33.98%, 11.87% and 9.64%, respectively.

Regarding the measured data for the diameter of spheres, Table 3 shows that, while for the larger spheres (Ø 18 and Ø 25 mm) the pre-sandblasting results were lower than the nominal value, for the Ø 10 mm sphere the average value was higher than the nominal value. 

However, the comparison before and after sandblasting provides values much closer to the reference (contact) values. This can be considered a success of the sandblasting process, which, by eliminating brightness, allows the laser to measure diameters much closer to the reference ones, even compensating differences as large as 0.116 and 0.164 in spheres of Ø 18 and Ø 25 mm, respectively. A comparison of the form deviation data before and after sandblasting is also shown in Table 3. Initially, the laser measurement averaged large form deviations, on the order of 0.15 to 0.19 mm. However, once the spheres are sandblasted, the laser measurements also offer very sharp improvements, ranging from 0.092 to 0.123 mm, leaving the form deviations at values in the range of 0.052 to 0.068 mm, also closer to the reference values.

With the laser equipment available, two types of improvements could be contrasted. On the one hand, the improvement in the density and coverage of the point cloud. This improvement was evident, since the coverage with pre-sanded spheres was very poor. In fact, in some cases (Ø 10 mm spheres), not all spheres could be correctly reconstructed. Consequently, the pre- and post-sandblasted comparison was only possible on all spheres when using high gain.

**Figure 7:**
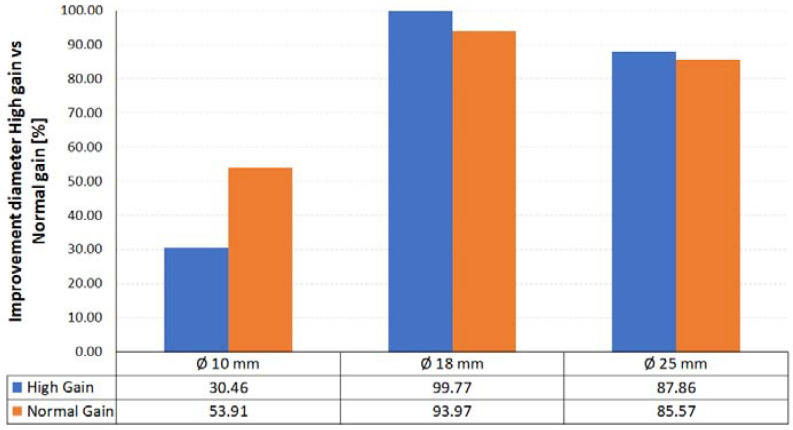
Diameter value improvements in non-contact measurement with medium and high gain in all three diameters.

The second improvement achieved is related to the dimensional approach of the laser measurements to the CMM measurements (reference). Meaning by improvement the relation (%) between the parameter measured by laser with respect to the parameter measured by contact. In other words, a 100% improvement in any of the measurements would mean that the laser obtains the same measurement as the CMM by contact. Thus, Figure 7 shows the improvements obtained in the values of the diameters, both in high gain and normal gain. The improvements are substantial, although not homogeneous, in all the range of diameters. An even greater improvement is noted in the large spheres, Ø 18 and Ø 25 mm, where the improvements are very high (>85%). The diameters obtained are very close to the reference values of the spheres, and even more so at high gain.

**Figure 8:**
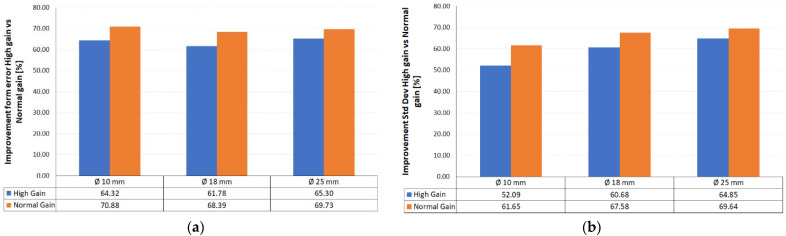
Improvements with medium- and high-gain laser measurements: (**a**) in form deviation; (**b**) in standard deviation.

Regarding the form deviation, the average improvement was 63.80% in high-gain mode and 69.67% in normal-gain mode. On the other hand, in the data relating to the standard deviation of the point cloud, an average percentage improvement of 59.21% was observed with the sensor filter at high gain and 66.29% at normal gain. Both parameters refer to measurements obtained on the spheres in a post-sandblasted state. In any case, homogeneous improvement values are observed regardless of the size of the sphere considered (Figure 8).

The standard deviation value is one of the parameters that best characterizes the quality of a point cloud [5], especially when considering its approximation to a mathematically well-defined geometry, as is the case of the sphere. This value is even a good substitute for metrological form deviation (ISO 1101:2017), measuring how good the point cloud is when fitting to a perfect sphere.

**Figure 9:**
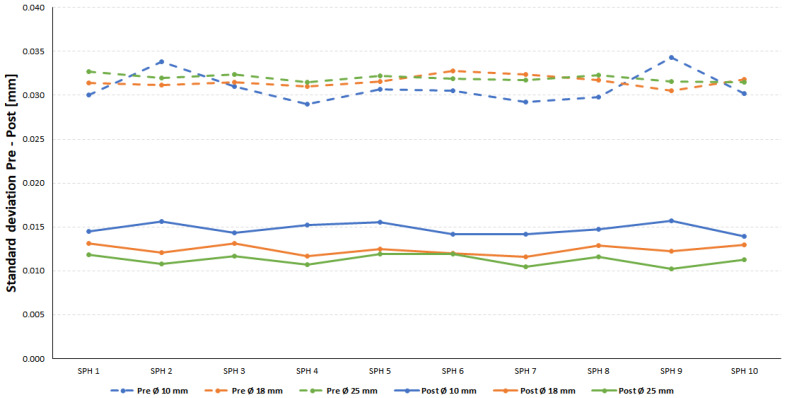
Standard deviation values pre- and post-sandblasting (laser measurements).

As a summary, and using the standard deviation value as a measure of the quality of the cloud fit, the graph in Figure 9 was produced. Dashed lines show the standard deviation measurements on original spheres, while solid lines show the standard deviation measurements of the laser clouds on sandblasted spheres.

Here the qualitative leap that the sandblasting process generates on the laser measurements (at high gain) can be clearly observed. While the values of the standard deviation measured before the sandblasting process, oscillate between 0.029 and 0.034 mm, after the sandblasting process, the standard deviations have decreased substantially (between 0.010 mm and 0.015 mm).

Moreover, the graph also shows that, for any range of diameters considered, the “vertical” oscillation range in the standard deviation is very small. This gives an idea of the goodness of the sandblasting process. Despite being a manual process, it achieved a fairly uniform distribution on all the spheres of each plate, regardless of the position of each sphere within the plate.

## 4. Conclusions

The pre- and post-sandblasting comparison of the spheres by contact and non-contact (laser triangulation sensor) measurement provides interesting data on the modification suffered by the sphere surfaces. In the analysis, the use of a wide number of spheres (three sets of 10 spheres) ensured the repeatability of the process. 

The sandblasting process has affected the surface roughness from *Ra* < 0.1 µm to *Ra* = 0.5~0.6 µm for all the spheres, indicating that this parameter has little influence from a dimensional and geometrical point of view. Apart from roughness, three parameters were considered for the study: the sphere diameter, the form deviation (sphericity) and the standard deviation of the point cloud with respect to the best fit sphere. The first two measurands are perfectly defined from a metrological point of view, while the third (standard deviation) is preferable as an indicator of the form deviation of the surface for high density point clouds.

As a first conclusion, the effect of sandblasting on the spheres is acceptably small, with minimal changes in diameter (2.7 µm) and, more importantly, in form deviation (1.7 µm). These characteristics validate the use of these spheres as reference artifacts for calibrating optical equipment, whose estimated accuracies are in the order of 25 µm to 40 µm (or even more). 

As a second conclusion, and now derived from the laser measurements, it can be observed that the increase in the density of the point cloud after sandblasting the spheres is very remarkable. So much so that, before sandblasting and at normal gain, the laser sensor was not able to capture clouds with good coverage. This defect was much more pronounced in small spheres. Even in the case of high gain, the improvements in the quality of the point cloud are considerable, with values of improvement in the order of 60 to 70%, both in terms of diameter and form deviation obtained with the best-fit cloud. Therefore, this type of surface finish can be considered as a good solution for the application of these spheres as reference artefacts in GD&T measurements with laser triangulation equipment.

As for the analysis performed with the standard deviation parameter, it can be seen that the value of the standard deviation between the point cloud of the pre-sandblasted and post-sandblasted sphere drops by almost half. That is, the fit of the point cloud to a sphere (value similar to the form deviation) is twice as good (almost 100% improvement) when sandblasted spheres are used. It can be stated that the brightness elimination of the spheres did not involve an important loss of sphericity that, on the contrary, remained within acceptable limits of accuracy.

This study demonstrates that sandblasted spheres, with average sphericity lower than 0.005 mm can be used as reference elements for non-contact measurement equipment with accuracies in the order of 0.040~0.050 mm. The validity of this statement is further supported by the low cost of the finishing process (manual sandblasting) and by the low cost of the stainless-steel spheres commonly used in industrial bearings.

## Figures and Tables

**Table 1:** Measurement results of the original (pre-sandblasted) spheres.

Sphere Size	Average Diameter [mm]			Average Form Deviation [mm]			Average Best-fit Sphere Std. Dev. (Laser)	Average No. Points (Laser)
	CMM	Laser	Difference	CMM	Laser	Difference		
Ø 10 mm	10.0026	10.0137	0.0111	0.0035	0.1908	0.1873	0.0309	10,431
Ø 18 mm	17.9977	17.8846	−0.1132	0.0023	0.1501	0.1478	0.0316	38,485
Ø 25 mm	25.0049	24.8608	−0.1441	0.0028	0.1492	0.1464	0.0320	73,079

**Table 2:** Comparison of contact measurement results regarding diameter and form deviation pre- and post-sandblasting.

Sphere Size	Average Diameter [mm]			Average Form Deviation [mm]		
	Pre	Post	Difference	Pre	Post	Difference
Ø 10 mm	10.0026	10.0054	0.0028	0.0035	0.0046	0.0010
Ø 18 mm	17.9977	18.0004	0.0027	0.0023	0.0044	0.0021
Ø 25 mm	25.0049	25.0075	0.0026	0.0028	0.0047	0.0020

**Table 3:** Comparison of laser measurement results regarding diameter and form deviation pre- and post-sandblasting.

Sphere Size	Average Diameter [mm]			Average Form Deviation [mm]			Average No. Points
	Pre	Post	Difference	Pre	Post	Difference	
Ø 10 mm	10.0137	9.9976	−0.0161	0.1908	0.0681	−0.1227	13,975
Ø 18 mm	17.8846	18.0006	0.1161	0.1501	0.0574	−0.0927	43,052
Ø 25 mm	24.8608	25.0250	0.1642	0.1492	0.0518	−0.0974	80,126

## Data Availability

Not applicable.
